# Management of post cardiac transplantation immunosuppression and COVID-19: A case report

**DOI:** 10.1016/j.amsu.2021.102875

**Published:** 2021-09-21

**Authors:** Imane Melhaoui, Younes Oujidi, Inass Arhoun El Heddad, Amine Bensaid, Houssam Bkiyar, Yassamine Bentata, Brahim Housni

**Affiliations:** a Nephrology Department, Mohammed VI University Hospital, Faculty of Medicine and Pharmacy, Oujda, Morocco; b Anaesthesia and Resuscitation Service – Hospital University Mohammed VI of Oujda. Oujda Faculty of Medicine and Pharmacy, Mohammed Premier University, Oujda, Morocco

**Keywords:** Heart transplant, COVID-19, SARS-CoV-2, Immunosuppressors

## Abstract

**Background:**

There is very limited experience in management of heart transplant (HT) recipients and their immunosuppressive drug therapies while confronted with a SARS-CoV-2 infection.

**Case details:**

We report the case of a 60-year-old male, heart transplant recipient patient, admitted in our ICU for severe COVID-19. His immunosuppressors were discontinued. He presented an ARDS, a multiple organ failure and a refractory septic shock that eventually resulted in his death.

**Discussion:**

Multiple studies reported a lower incidence of SARS-Cov-2 infection in HT recipients compared to the general population, probably due to their prior knowledge and use of protective and barrier measures; but when infected they tend to have poorer outcomes and higher fatality; on account of their pre-existing comorbidities and immunodeficiency. Therefore, the management of the immunosuppressive therapy raises a challenge, in the absence of trials. Physicians rely on experts’ recommendations, to maintain the immunosuppressors in case of mild COVID-19, lower to the bare minimum or even discontinue them in case of critical COVID-19 or systemic complications.

**Conclusion:**

COVID-19 infection is associated with poor outcomes and high mortality in HT recipients, and their immunosuppressive therapy management still raises questions and challenges in the absence of trial-validated data.

## Highlights

• Heart transplant recipients are believed to be at a higher COVID-19 infection risk.
• ICU admission rate and the higher mortality risk is due to pre-existing comorbidities and immunodeficiency status of patients.

## 1 Introduction

Since its emergence in December 2019, the Coronavirus disease (COVID-19) has caused a global pandemic and resulted in a substantial worldwide morbidity and mortality.

The resulting major health crisis has caused an unprecedented effect on various diseases and their management, especially in the transplantation field; and with relatively limited data and knowledge on SARS-CoV-2 effects on the body, many questions remain unanswered. Post-transplant patients are considered fragile and therefore more likely to be affected by the COVID-19; by not only their immunosuppression treatment, but also, their comorbidities and further contact with hospitals and Health workers[1]. The balance between a COVID-19 infection and its rapid progression under an immunosuppressor treatment and the immunodeficiency it causes and the possible rejection of the transplanted organ is the principal dilemma professionals are exposed to Ref. [2].

We report the case of a heart transplant patient infected with the SARS-CoV-2 treated for a COVID-19 pneumopathy at our intensive care unit.

This case report has been reported in line with the SCARE Criteria [10].

### 1.1 Clinical case

This is a 60-year-old male patient, with hypertension and diabetes mellitus under treatment. He underwent heart transplantation on 2017 in Spain, and currently under immunosuppressors: Tacrolimus 1mg and Mycophenolate mofetil 0.5mg, twice daily.

He reported a recent trip to Spain two weeks before the symptoms for a check-up with his doctors, but had no contact with any known-SARS-CoV-2 infected individuals.

The patient had a history of fever, runny nose, sore throat and dry cough evolving for 8 days prior to his admission. His evolution worsened and he developed dyspnea and chest pain, motivating his visit to the emergency room.

Clinically, the patient was conscious, hemodynamically stable, with an oxygen saturation of 78% in ambient air; 96% under 15L oxygen with a high concentration mask. He also had a normal body temperature.

Biologically, he had a lymphocytopenia at 220 elements/m3, a normal troponin level of 17 ng/mL, with positive inflammation markers: C-reactive protein level at 106 mg/L, Ferritin level 679 mg/L; fibrinogen level at 8.7 mg/L, Lactic dehydrogenase (LDH) level at 370 mg/L. A SARS-CoV-2 reverse transcriptase PCR was performed and came back positive.

Arterial blood gas was performed and showed a hypoxemia with a PaO2 at 41 mmHg.

He also benefitted from a thoracic CT-scan with an extent of pulmonary damage of 75%, and a cardiac evaluation with a normal transthoracic echocardiography.

A non-invasive ventilation based on High Flow Nasal Cannula (HFNC) was started at the 2end day of his admission; the immunosuppressors were discontinued and he was put under: Azithromycin, Vitamin C 2g/day, Zinc 45mg/day, Enoxaparin 0.6UI/12h, Dexamethasone 6mg/day; Aspirin 160mg/day and antibiotics: 3rd generation Cephalosporins 2g/day and quinolones (Ciprofloxacin 200mg/12h). He also received two doses of Tocilizumab (400mg with a 10 day interval).

His antibiotics were changed to Piperacillin-Tazobactam 4g/8h, Amikacin 1,5g/day and Voriconazole 200mg/12h when nosocomial infection was confirmed by culture.

The patient unfortunately presented complications, such as acute respiratory distress syndrome in the 3 erd day of admission, and was intubated and put under intensive mechanical ventilation. The progression of the disease was very fast for his case He presented a multi-organ failure (acute kidney injury and disseminated intravascular coagulation) and a refractory septic shock in 2end days after intubation, that resulted in his death at one week of his admission.

## 2 Discussion

When confronted with this new COVID-19 pandemic, clinicians found themselves challenged while facing immunocompromised transplant recipients and their SARS-CoV-2 infection; and how their immunosuppressors intake's role in the systemic inflammatory response[2].

Two meta-analysis conducted in Germany by Rivinius et al. and in China by Ren et al. both reported that a COVID-19 infection among heart transplant patients were rarely reported. This can be explained by these patients’ prior knowledge of hygiene requirements and barrier measures due to their immunodeficiency status [1,3].

However, when affected, heart transplant recipients are more likely to report a severe COVID-19 with higher ICU admission rate, increased mechanical ventilation need, and a higher mortality risk[3].

The effect of immunosuppressors therapy on the COVID-19 infection is nowadays still a matter of debate. In fact, in vitro data suggest that immunosuppressive therapy (such as Interferon α and Cyclosporine) might result in the inhibition of viral replication by altering the T-cell response [[3], [4], [5]] and have therefore a protective mechanism; however long-term immunosuppression is known to increase infection-risk [6].

Concerning the management of immunosuppressive therapy in a heart transplant recipient infected with SARS-CoV-2, no evidence-based data is validated. Strategies are based upon expert opinions and transplant societies recommendations, and these guidelines are based upon the severity of the COVID-19 illness:

- If mild COVID-19 (no dyspnea or hypoxemia, oxygen saturation >94%); the immunosuppressors are maintained with a frequent monitoring for worsening symptoms[7,8].
- If the disease is moderate or severe, the patients must obviously be treated with in-hospital supportive care; many centers suggest keeping only the antiproliferative agents (such as Mycophenolate mofetil or Azathioprine) and to a bare minimum; with a monitoring of their lymphocyte count [7].
- If the disease becomes critical (complications such as respiratory failure requiring ventilation support or ECMO, circulatory collapse, acute kidney failure, cardiomyopathy …) all immunosuppressive drug therapy must be discontinued [8].

Corticosteroids such as (dexamethasone 6mg/d up to 10 days) can be instituted, to avoid adrenal insufficiency [7] and for being associated to survival improvement in patients under ventilation support [9].

## 3 Conclusion

Heart transplant recipients are believed to be at a higher COVID-19 infection risk and associated with a higher ICU admission rate, more severe COVID-19, and higher mortality risk, due to their pre-existing comorbidities and their immunodeficiency status.

Regarding maintaining or discontinuing immunosuppressive drug therapy we can only depend on expert opinions, in the wait of future investigations and trial-validated data.

Strong efforts must be carried out to control the pandemic spread and avoid its poor outcomes especially in the absence of a cure.

## Competing interests

The authors declare that they have no competing interests.

## Funding

The authors declare that they have no funding.

## Provenance and peer review

Not commissioned, externally peer reviewed.

## Please state any conflicts of interest

No conflicts Of Interest.

## Please state any sources of funding for your research

No Source Of funding.

## Ethical approval

This is a case report that does not require a formal ethical committee approval. Data were anonymously registered in our database. Access to data was approved by the head of the department.

## Consent

Written informed consent was obtained from the patient for publication of this case report and any accompanying images. A copy of the written consent is available for review by the Editor-in-Chief of this journal.

## Author contribution

Dr. Iman Melhaoui: are the principal investigators that collected and analyzed data, wrote the manuscript and prepared the final draft for the submission. Dr. Amine Bensaid, Dr Oujidi Younes, and Dr. Inass Arhoun EL Heddad: participate in patients’ management. Prof. Brahim Housni, Prof. Yassamine Bentata and Prof. Houssam Bkiyer: supervised the research project and approved the final draft for publication All authors approved the final version of the manuscript.

## Registration of research studies

This is a case report that does not require a formal ethical committee approval. Data were anonymously registered in our database. Access to data was approved by the head of the department.

## Guarantor

Dr Imane Melhaoui and Dr Oujidi Younes.

## Declaration of competing interest

None.
